# “Low‐Positive” MOG‐IgG Cases Among Adults With a First Event Suggestive of Multiple Sclerosis

**DOI:** 10.1002/acn3.70496

**Published:** 2026-08-21

**Authors:** Javier Villacieros‐Álvarez, Carmen Espejo, Georgina Arrambide, Mireia Castillo, Marta Rodriguez, Helena Ariño, Iker Elosua, Carmen Tur, Angela Vidal‐Jordana, Andreu Vilaseca, Joaquín Castilló, Ingrid Galán, Luciana Midaglia, Carlos Nos, Breogan Rodríguez‐Acevedo, Ana Zabalza, Luca Bollo, Agustín Pappolla, René Carvajal, Neus Mongay‐Ochoa, Jordi Rio, Jaume Sastre‐Garriga, Manuel Comabella, Gesualdina Busiello, Sofia Sceppacuercia, Àlex Rovira, Xavier Montalban, Mar Tintoré, Cristina Auger, Álvaro Cobo‐Calvo

**Affiliations:** ^1^ Neurology Department and Centre D'esclerosi Múltiple de Catalunya (Cemcat) Hospital Universitari Vall D'hebron, Vall D'hebron Institut de Recerca. Barcelona Spain; ^2^ Universitat Autònoma de Barcelona Barcelona Spain; ^3^ Neurology Department, Unidad de Neuroinmunología Hospital Universitari Germans Trias i Pujol. Badalona Spain; ^4^ Neurology Department Hospital de la Santa Creu i Sant Pau Barcelona Spain; ^5^ Section of Neuroradiology, Department of Radiology Hospital Universitari Vall D'hebron. Vall D'hebron Institut de Recerca Barcelona Spain; ^6^ Universitat de Vic‐Central de Catalunya (UVic‐UCC) Vic Spain

**Keywords:** MOGAD, MOG‐IgG, multiple sclerosis, low‐positive

## Abstract

**Objective:**

To determine the prevalence and clinical characteristics of patients with “low‐positive” (LP) MOG‐IgG (titres 1:160–1:320) among adults with a first demyelinating event (FDE) suggestive of multiple sclerosis (MS).

**Methods:**

From the Barcelona CIS inception cohort, we included adult patients with serum collected ≤ 6 months from the FDE. MOG‐IgG was assessed by a cell‐based assay with flow cytometry (CBA‐FC). Demographic, clinical, and paraclinical data were compared among seronegative, LP, and “clear‐positive” (CP; ≥ 1:640) patients. Supporting MOGAD features and final diagnoses were retrospectively reviewed in seropositive cases.

**Results:**

Of 613 patients, 42 (6.9%) were MOG‐IgG positive (CP = 17; LP = 25). LP patients were indistinguishable from seronegative patients but differed from CP patients, who more frequently had optic neuritis, lacked cerebrospinal fluid‐oligoclonal bands, and were less likely to meet the McDonald criteria (*p* < 0.05). At the last follow‐up, 64% of LP versus 18% of CP patients were diagnosed with MS (*p* = 0.004). Only one LP patient fulfilled MOGAD criteria, compared with 14 CP patients (*p* < 0.001).

**Interpretation:**

Lowering the CBA‐FC positivity threshold to ≥ 1:160 had limited diagnostic yield in this MS‐predominant cohort, as most LP cases were ultimately diagnosed with MS. These findings support cautious interpretation of LP MOG‐IgG results in this clinical setting.

## 1 Introduction

The diagnosis of myelin oligodendrocyte glycoprotein (MOG) antibody‐associated disease (MOGAD) primarily relies on the detection of MOG‐IgG in serum by a cell‐based assay (CBA) in the setting of a core clinical demyelinating event, together with the exclusion of alternative diagnoses, especially multiple sclerosis (MS) [1] MOG‐IgG titres are crucial for an accurate diagnosis, as low titres have been associated with a higher risk of false‐positive results [2]. In order to overcome this limitation, the criteria required for additional clinical or radiological supporting features to make the diagnosis of MOGAD in MOG‐IgG low positive (LP) titres. However, such supporting information is not necessary in cases with “clear‐positive” (CP) results according to the 2023 criteria [1].

Definitions of CP and LP are not widely standardized and depend on the assay used in each laboratory. Specifically, some specialized centers using a live CBA with flow cytometry (CBA‐FC) only consider a CP result as part of the criteria, with a screening dilution of 1:640, but without the possibility of applying supporting information to LP cases (lower dilutions). In a recent study, we identified 17 MOG‐IgG CP cases among 630 adult patients presenting with a first demyelinating event (FDE) suggestive of MS with CBA‐FC, of whom 14 were ultimately diagnosed with MOGAD [3] Nonetheless, it remains uncertain whether “true” MOGAD cases at lower antibody titres might have been missed.

In addition, among centers employing CBA‐FC, the interpretation of MOG‐IgG results is inconsistently based on either qualitative assessment or quantitative measures (e.g., delta [Δ] or ratio of fluorescence intensity [FI]), often using arbitrary cut‐offs or values derived from control populations [1, 4, 5].

In this context, we aimed to determine the frequency and clinical characteristics of LP cases among adult patients presenting with an FDE suggestive of MS, and to explore the relationship between qualitative and quantitative MOG‐IgG measurements.

## 2 Methods

### 2.1 Study Design and Participants

This study was based on the Barcelona clinically isolated syndrome (CIS) inception cohort at the Multiple Sclerosis Center of Catalonia, Vall d'Hebron University Hospital, Barcelona [6]. This cohort included 1345 patients prospectively enrolled between January 1995 and August 2020, with the following inclusion criteria: (1) a first demyelinating event suggestive of MS, (2) age between 18 and 50 years at disease onset, and (3) the first clinical evaluation within 3 months of disease onset.

The first demyelinating event suggestive of MS was defined as a first clinical episode reflecting a focal or multifocal inflammatory demyelinating event in the central nervous system, developing acutely or subacutely, with a duration of at least 24 h, in the absence of fever or infection. Patients with diagnoses other than MS and MOGAD (i.e., AQP4‐Ab‐positive patients, acute demyelinating encephalomyelitis [ADEM], autoimmune encephalitis, connective tissue diseases, infectious or metabolic diseases) confirmed at any time during the follow‐up were excluded from the cohort.

Patients with available serum samples collected within 6 months from the disease onset were finally included in this study.

### 2.2 Clinical Information

Demographic and clinical features prospectively collected in the Barcelona inception cohort routinely include sex, age at the first demyelinating event, topography at disease onset (optic nerve [optic neuritis (ON)], spinal cord, infratentorial, or multifocal), presence and dates of relapses, and disability according to the Expanded Disability Status Scale (EDSS). EDSS scores are obtained within 3 months after the first demyelinating event and at least annually during stability periods or in case of worsening. A relapse is defined as the occurrence of new symptoms or exacerbation of existing symptoms persisting for at least 24 h in the absence of fever and infection. Relapses and EDSS are assessed by appropriately trained neurologists specializing in MS.

Laboratory data include the presence of cerebrospinal fluid (CSF)‐restricted oligoclonal bands (CSF‐OBs) in all patients who underwent a lumbar puncture.

Standardized brain and spinal cord magnetic resonance imaging (MRI) scans were performed according to recommendations [7].

Brain MRI information includes the number (0, 1–3, 4–8, > 8) of T2 lesions, the presence of gadolinium (Gd)‐enhancing lesions (no/yes), and the new T2 lesion number at last follow‐up. Lesion number (0, 1, 2–3, > 3) and the presence of Gd‐enhancement on spinal cord MRIs are also collected. Dates of fulfillment of the 2017 McDonald criteria were collected, defining baseline as the first year after the first demyelinating event [8].

Treatment information includes the dates of initiation and discontinuation, and the type of treatment: acute therapy such as corticosteroids and maintenance therapy.

In addition to prospective standardized data collection, supporting clinical and radiological features of MOGAD [1] during all attacks were retrospectively recorded in MOG‐IgG‐positive (CP and LP) patients by reviewing the hospital's medical records and MRI scans. Furthermore, in patients who fulfilled both MS and MOGAD diagnostic criteria at their last follow‐up, clinical information and MRI scans were comprehensively reviewed by two neurologists (J.V‐A, A.C‐C) and one radiologist (C.A.), all with expertise in neuroimmunological diseases and blinded to MOG‐IgG titres. Any discrepancies were resolved through a multidisciplinary meeting to reach a consensus on the final diagnosis.

### 2.3 Antibody Assessment

Serum samples from patients and controls were collected and stored at −80°C for research purposes. MOG‐IgG was assessed by a live‐CBA‐FC as previously described [3], using an initial dilution of 1:160 with further titration. Twenty healthy controls (HC) and 11 controls with other non‐inflammatory neurological diseases (ONIND) were also assessed for MOG‐IgG. All the patient cohort was assessed for AQP4‐IgG as previously described [3].

The CP group was defined as patients with titres of ≥ 1:640 and the LP group as patients with titres between 1:160 and 1:320.

### 2.4 Quantitative Analyses of FI

Quantitative assessment of MOG‐IgG binding was performed at a serum dilution of 1:640. The analysis involved the following steps: (1) gating MOG‐transfected and non‐transfected cells separately for each well; (2) extracting the mean, median, and geometric mean FI values for both gated populations; (3) calculating three quantitative measures: ΔMOG (difference in FI between transfected and non‐transfected cells [FI transfected−FI non‐transfected]), ratio‐MOG (FI transfected/FI non‐transfected), and delta‐ratio‐MOG ([FI transfected−FI non‐transfected]/FI non‐transfected). Positive thresholds for each formula were established by calculating 2, 3, 4, 5, and 6 standard deviations (SD) above the mean FI values obtained from the control cohort.

### 2.5 Statistical Analyses

The frequency of CP and LP MOG‐IgG in the whole cohort was determined, and the clinical and demographic characteristics among seronegative, LP, and CP groups were compared using the *χ* ^2^ test (or Fisher's exact test) for categorical variables and the Kruskall–Wallis test for continuous variables, as appropriate. Descriptive qualitative and quantitative information is given as percentages, median, interquartile range [IQR], and range.

Time to fulfillment of the McDonald 2017 criteria was compared across the three groups with Kaplan–Meier curves (log‐rank test) as well as Cox regression analysis. Results are given in hazard ratios (Confidence interval [CI] 95%).

In order to select the best quantitative cutoff discriminating MOG‐IgG ≥ 1:640 vs. < 1:640 two different analyses were performed:

Analysis 1: The correlation between qualitative MOG‐IgG titres and quantitative FI measures was assessed using the Spearman rank correlation test (Rho) in CP patients. Then, sensitivity and specificity analyses were performed by using the different positivity thresholds of the most correlated formula according to the mean + SD of controls to discriminate between MOG‐IgG ≥ 1:640 and < 1:640.

Analysis 2: The Youden index was used in the total patient cohort to define the best cutoff value of the formula to discriminate between MOG‐IgG ≥ 1:640 vs. < 1:640.

### 2.6 Standard Protocol Approvals, Registrations, and Patient Consents

The study was approved by the Clinical Research Ethics Committee at Vall d'Hebron University Hospital (EPA [AG]57/2013 [3834]). All patients signed written informed consent.

## 3 Results

### 3.1 Prevalence of MOG‐IgG and Clinical Characteristics According to Antibody Status

**FIGURE 1:**
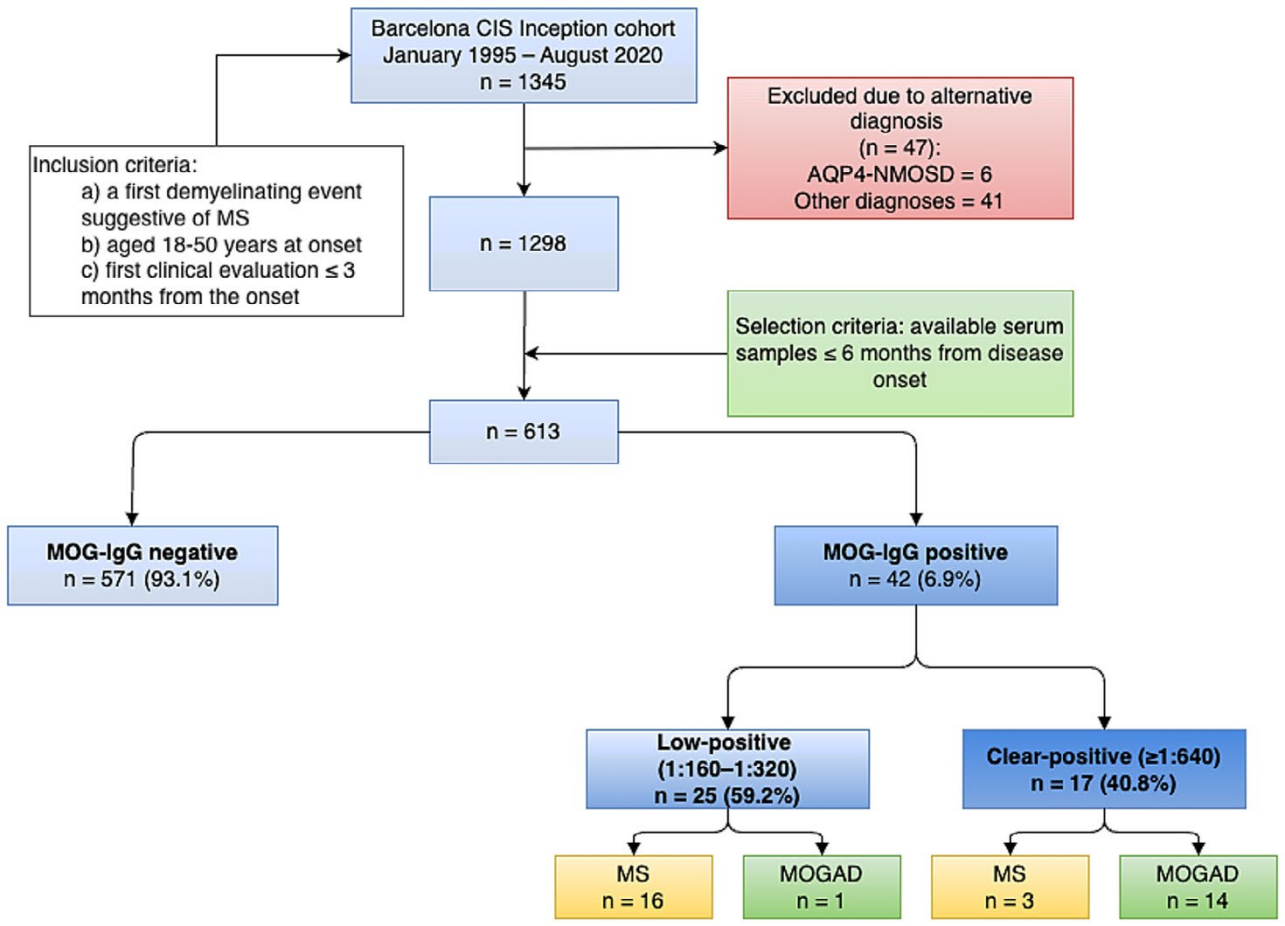
Flow chart of the cohort selection and MOG‐IgG results. CIS, clinically isolated syndrome; MS, multiple sclerosis; AQP4‐NMOSD, aquaporin‐4 neuromyelitis optica spectrum disorder; MOG, myelin oligodendrocyte glycoprotein; MOGAD, MOG‐IgG‐associated disease.

Among the 613 patients included, 42 (6.85%) were positive for MOG‐IgG (CP, *n* = 17 [2.77%]; LP, *n* = 25 [4.08%]). (Figure 1) The 31 controls had a negative MOG‐IgG result. All 613 patients were negative for AQP4‐IgG. The median (IQR) titres of the 42 MOG‐IgG positive cases were 240 (160–1280); for the CP group, 2560 (1280–10,240); and for LP group, 160 (160–160).

During follow‐up, most LP patients (16/25 [64.0%]) were ultimately diagnosed with MS, whereas most CP patients (14/17 [82.4%]) had MOGAD.

**FIGURE 2:**
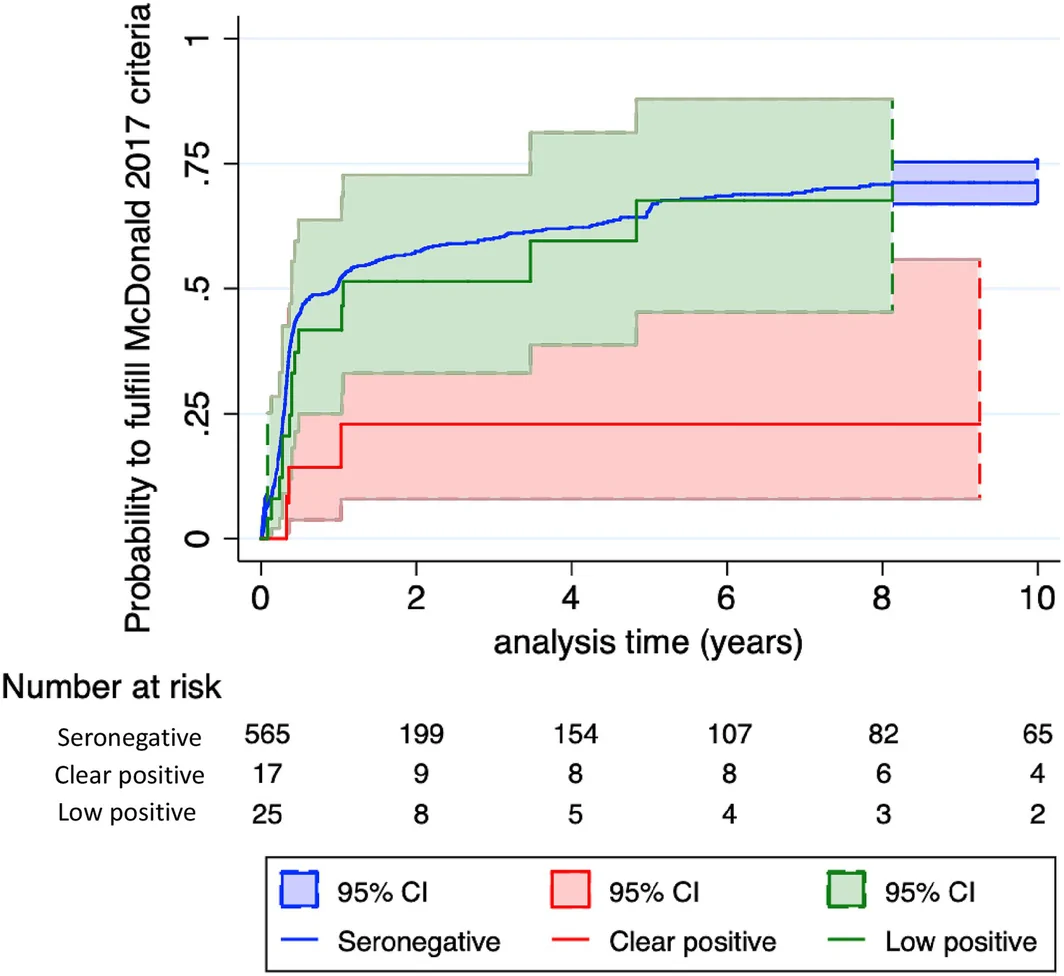
Kaplan Meier curves showing time to McDonald 2017 fulfillment in seronegative, low‐positive, and clear‐positive MOG‐IgG patients.

Specifically, 380 (66.6%) seronegative and 16 (65%) LP patients fulfilled the 2017 McDonald criteria, compared to only 3 (17.7%) CP patients (*p* < 0.001). (Table 1; Figure 2).

**TABLE 1:** Baseline and follow‐up characteristics of seronegative, “low‐positive” and “clear‐positive” patients.

Variables	Negative patients (*n* = 571)	LP patients (*n* = 25)	CP patients (*n* = 17)	*p*‐value overall	*p*‐value negative vs LP	*p*‐value negative vs CP	*p*‐value LP vs CP
MOG‐IgG titres; median (IQR)		160 (160–160)	2560 (1280–10,240)				
Sex female; *n* (%)	390 (68.3)	18 (72.0)	12 (70.6)	0.859	0.672	0.838	0.859
Age onset; median (IQR) years	33.2 (26.9–39.7)	29.9 (26.1–35.1)	32.5 (23.6–35.9)	0.334	0.247	0.336	0.990
Topography onset; *n* (%)							
Optic nerve	209 (36.6)	11 (44.0)	14 (82.4)	0.078	0.526	**< 0.001**	**0.024**
Spinal cord	156 (27.3)	7 (28.0)	1 (5.9)	0.129	1.000	0.052	0.114
Infratentorial	134 (23.5)	5 (20.0)	1 (5.9)	0.242	0.813	0.139	0.374
Multifocal	72 (12.6)	2 (8)	1 (5.9)	0.801	0.757	0.709	1.000
EDSS onset; median (IQR)	2 (1–3)	2 (1–2.5)	2 (1–3)	0.640	0.660	0.415	0.328
CSF‐OBs; *n* (%)	345 (61.7)	11 (44.0)	2 (12.5)	**< 0.001**	0.076	**< 0.001**	**0.045**
Radiological information							
Normal brain MRI; *n* (%)	142 (26.1)	8 (32.0)	9 (56.3)	**0.029**	0.509	**0.007**	0.124
> 8 T2 lesions; *n* (%)	264 (48.4)	9 (36.0)	3 (18.8)	**0.032**	0.223	**0.007**	0.305
Brain Gd‐enhanced lesions; *n* (%)	308 (53.9)	14 (56.0)	8 (47.1)	0.882	0.840	0.575	0.569
Spinal cord lesions; *n* (%)	123/345 (35.6)	5/16 (31.3)	2/11 (18.2)	0.527	0.719	0.232	0.446
Time onset‐sampling; median (IQR) days	24 (2–62)	29 (14–56)	41 (15.5–53)	0.869	0.924	0.605	0.643
Acute treatment within 1‐month prior sampling; *n* (%)	180/223 (80.7)	6/7 (85.7)	7/8 (87.5)	1.000	1.000	1.000	1.000
Follow‐up							
FU duration; median (IQR) years	8.9 (4.3–15.8)	8.0 (2.1–13.5)	6.7 (1.0–10.6)	0.095	0.212	0.069	0.582
ARR; median (IQR)	0.0 (0.0–0.18)	0.0 (0.0–0.11)	0.0 (0.0–0.0)	0.084	0.221	0.058	0.356
Final EDSS; median (IQR)	1.5 (1.0–2.0)	1.0 (0.0–1.5)	1.0 (0.0–2.0)	0.184	0.205	0.170	0.673
Maintenance treatment; *n* (%)	301 (52.7)	14 (56.0)	4 (8.3)	**< 0.001**	0.839	**< 0.001**	**< 0.001**
New lesions on brain MRI; *n* (%)	284/510 (55.7)	10/22 (45.5)	3/13 (23.1)	**0.041**	0.345	**0.024**	0.282
Accumulated T2 lesions; median (IQR)	1 (0–6)	0 (0–6)	0 (0–0)	0.050	0.409	**0.017**	0.135
McDonald 2017 fulfillment; *n* (%)	380 (66.6)	16 (65.0)	3 (17.7)	**< 0.001**	0.472	**< 0.001**	**0.004**

*Note:* Bold values indicate statistically significant differences (*p* < 0.05).

Abbreviations: ARR, annualized relapse rate; CP, clear‐positive; CSF‐OBs, cerebrospinal fluid‐restricted oligoclonal bands; EDSS, expanded disability status scale; FU, follow up; IQR, interquartile range; LP, low‐positive; MRI, magnetic resonance imaging.

**FIGURE 3:**
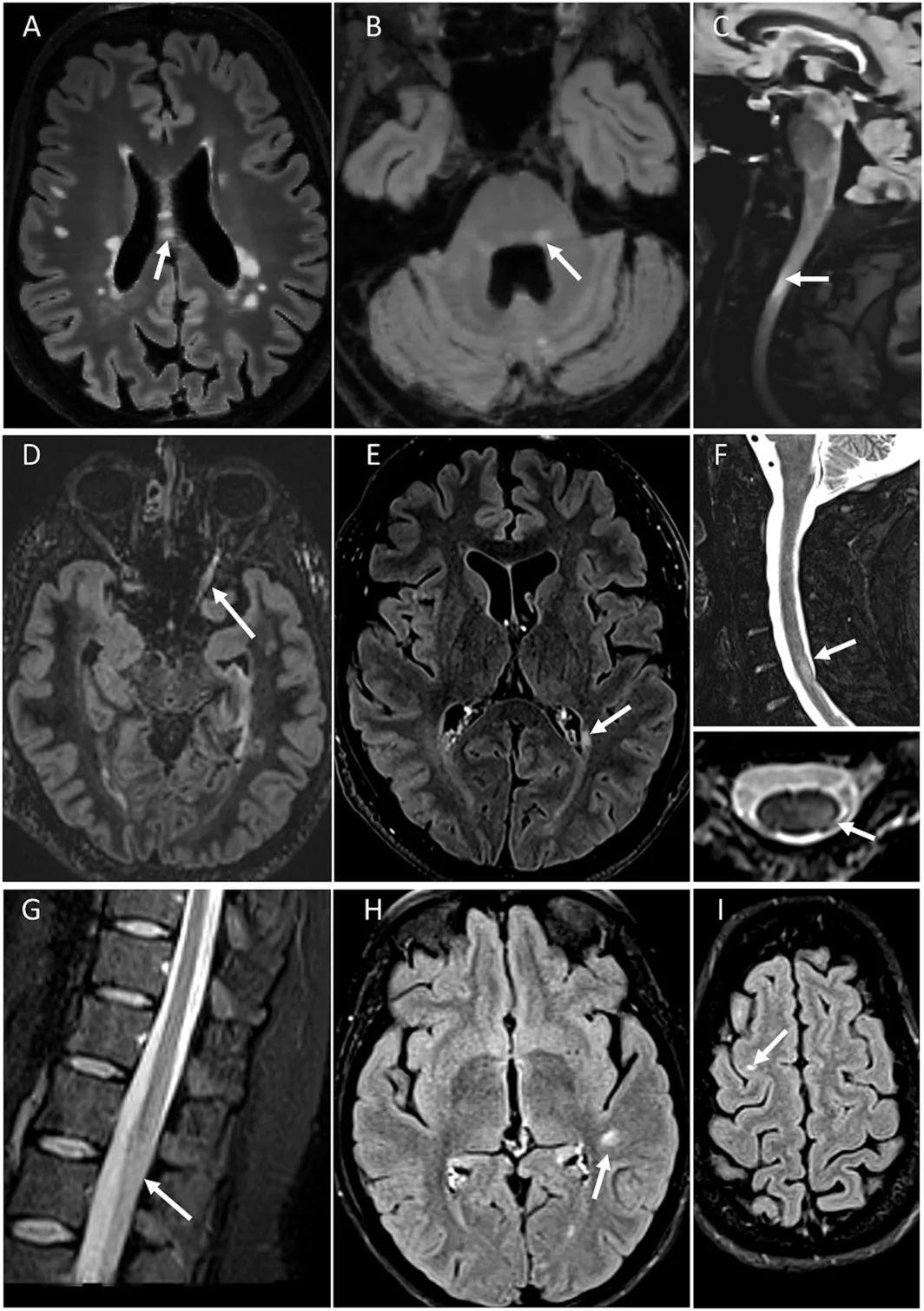
Supporting radiological features in low‐positive patients. Figure 3 shows MRI findings of three low‐positive patients (A‐C, a patient with typical MS lesions; D‐F: A patient with MS and a long optic nerve lesion on brain MRI; G‐I, a patient with MS and a conus lesion on spinal cord MRI).

Among the 16 LP patients meeting the 2017 McDonald criteria, three (18.8%) had at least one supporting clinical or MRI feature of MOGAD (one with disc edema, one with long ON lesion on brain MRI, one with a conus lesion on spinal cord MRI). However, all of them were ultimately diagnosed with MS. Clinical information for these three cases is detailed in the Supporting Information. The remaining 9 cases included: three monophasic isolated ON (one with disc edema who was diagnosed with MOGAD, and the other two remained as isolated ON since they did not have supporting features); one CRION (without supporting features); one isolated myelitis; one solitary demyelinating lesion in the temporal uncus presenting with hemianopsia; and three patients with neurological deficits but normal MRI and CSF (one brainstem syndrome, one cervical spinal cord syndrome, one hemispheric syndrome). Consequently, the final MOGAD criteria were met by 14/17 (82.4%) CP and 1/25 (3.8%) LP patients (*p* < 0.001). Among these 15 MOGAD cases, only one (6.7%) relapsed over a median (IQR) follow‐up of 6.67 (0.99–10.03) years. A description of the clinical features of LP cases is detailed in Table 2, and MRI findings in Figure 3.

**TABLE 2:** Clinical characterization of “low‐positive” MOG‐IgG cases.

	MOGAD (*n* = 1)	MS (*n* = 16)	Other (*n* = 8)
Clinical phenotype	Monophasic isolated unilateral ON	RR‐MS	Monophasic isolated unilateral ON (*N* = 2) CRION (*N* = 1) Monophasic isolated transverse myelitis (*N* = 1) Cerebral deficits with a solitary demyelinating lesion (*N* = 1) Neurological deficits without paraclinical findings (*N* = 3)
Female (%)	1 (100)	12 (75)	5 (62.5)
Age at onset, y (median, IQR)	23.2	30.3 (27–35)	30 (25.9–41.1)
Topography at onset	ON	7 ON, 5 spinal cord, 4 brainstem	3 ON, 2 spinal cord, 2 multifocal, 1 brainstem
EDSS at onset; median (range)	1.0	1.75 (0–4.0)	2.0 (0–3.0)
Presence of CSF‐restricted OBs, *n* (%)	0	11 (68.8)	0
Abnormal brain MRI, *n* (%)	0	16 (100)	2 (25.0)
Any clinical/MRI supportive feature at any attack, *n* (%)	Disc edema	3 (18.8) (1 disc edema, 1 long ON lesion, 1 conus lesion)	0
Chronic treatment, *n*	0	14 (8 IFN, 2 GA, 2 DMT, 2 clinical trials) 4 switched to HET (IFN➔ofatumumab, IFN➔fingolimod, IFN➔DMF, AG➔DMF)	0
Patients with relapses, *n* (%)	0	8 (50.0) (median 0.5 [0–6.0])	0
New T2‐lesions on follow‐up brain MRIs, *n* (%)	0	10 (66.7)	0
EDSS at last follow‐up; median (range)	0	1.5 (0.0–4.0)	0.0 (0.0–2.5)
Follow‐up duration, y (median, IQR)	8.1	9.9 (5.5–17.2)	1.7 (0.7–6.0)

Abbreviations: CRION, chronic relapsing inflammatory optic neuritis; CSF‐OBs, cerebrospinal fluid‐restricted oligoclonal bands; DMF, dimethyl fumarate; GA, glatiramer acetate; HET, high efficacy therapies; IFN, interferon; IQR, interquartile range; MOGAD, myelin oligodendrocyte antibody‐associated disease; ON, optic neuritis; RR‐MS, relapsing–remitting multiple sclerosis.

In keeping with the final diagnoses, LP patients were clinically indistinguishable from the seronegative group, with no differences in any of the clinical and paraclinical variables (Table 1). In contrast, CP patients more frequently presented with ON at onset, less frequently had CSF‐OBs, were less likely to fulfill the 2017 McDonald criteria at baseline, more frequently had a normal baseline brain MRI, and accumulated fewer new T2 lesions during follow‐up (all *p* < 0.05; Table 1).

Most seronegative and LP patients received maintenance treatment during the disease course (301 [52.7%] and 14 [56.0%], respectively, *p* = 0.839), in contrast to a lower proportion of CP patients (4 [8.3%], *p* < 0.001). (Table 1) Distribution of treatments among seropositive patients is depicted in Table S1. No differences in time from disease onset to sampling or in acute treatment pre‐sampling were found across the three groups. (Table 1).

### 3.2 Clinical and Radiological Supporting Features in MOG‐IgG Positive Patients

At the first attack, 11 (64.7%) CP patients had at least one clinical or MRI supporting feature of MOGAD compared to four (16.0%) LP patients (*p* = 0.003). Among the 24 seropositive patients presenting with ON and available clinical information, 9/14 (64.3%) CP patients, compared to 2/10 (20.0%) LP patients, had a supporting clinical feature (*p* = 0.047).

Bilateral ON was found in 2/14 (14.3%) CP patients compared to no LP patients, and disc edema was found in 7/13 (53.9%) CP patients compared to only 2/9 (22.2%) LP cases. Three (17.7%) CP patients had at least one MRI supporting feature at the first attack compared to two (8.0%) LP patients (*p* = 0.379) (Table 3).

**TABLE 3:** Supporting clinical and MRI features of MOGAD in “clear‐positive” and “low‐positive” patients at first attack and at any attack.

Supportive criteria	At first attack			At any attack		
	CP patients (*n* = 17)	LP patients (*n* = 25)	*p*	CP patients (*n* = 17)	LP patients (*n* = 25)	*p*
Any supporting clinical feature; *n* (%)	9/14 (64.3)	2/10 (20.0)	**0.047**	9/14 (64.3)	2/11 (18.2)	**0.042**
Bilateral optic neuritis; *n* (%)	2/14 (14.3)	0/10 (0.0)	0.493	2/14 (14.3)	0/11 (0.0)	0.487
Optic disc oedema; *n* (%)	7/13 (53.9)	2/9 (22.2)	0.203	7/14 (50.0)	2/11 (18.2)	0.208
Any supporting MRI feature; *n* (%)	3 (17.7)	2 (8.0)	0.379	4 (23.5)	2 (8.0)	0.202
Longitudinal optic nerve involvement; *n* (%)	2/12 (16.7)	0/18 (0)	0.152	3/12 (25.0)	1/18 (5.56)	0.274
Perineural optic sheath enhancement; *n* (%)	0/10 (0.0)	0/13 (0.0)	1.000	0/10 (0.0)	0/13 (0.0)	1.000
Longitudinally extensive transverse myelitis; *n* (%)	1/10 (10.0)	0/18 (0.0)	0.172	1/10 (10.0)	0/18 (0.0)	0.357
Central cord lesion or H sign; *n* (%)	1/10 (10.0)	0/19 (0.0)	0.161	1/10 (10.0)	0/19 (0.0)	0.345
Conus lesion; *n* (%)	0/8 (0.0)	1/16 (6.25)	1.000	1/8 (12.5)	1/16 (5.6)	0.529
Multiple ill‐defined T2 hyperintense lesions in white matter; *n* (%)	0 (0.0)	0 (0.0)	1.000	2 (11.8)	0 (0.0)	0.158
Deep gray matter involvement; *n* (%)	1 (5.9)	0 (0.0)	0.220	1/17 (5.9)	0 (0.0)	0.405
Ill‐defined T2‐hyperintensity involving pons, middle cerebellar peduncle or medulla; *n* (%)	2 (11.8)	1/23 (4.4)	0.565	3/17 (17.7)	1/23 (4.4)	0.069
Cortical lesion; *n* (%)	0 (0.0)	0 (0.0)	1.000	0 (0.0)	0 (0.0)	1.000
Any supporting clinical or MRI features; *n* (%)	11 (64.7)	4 (16.0)	**0.003**	12 (70.6)	4 (16.0)	**0.001**
Fulfillment of MOGAD criteria; *n* (%)	14 (82.3)	1 (3.8)	**< 0.001**	14 (82.3)	1 (3.8)	**< 0.001**

*Note:* Bold values indicate statistically significant differences (*p* < 0.05).

Abbreviations: CP, clear‐positive; LP, low‐positive; MOGAD, myelin oligodendrocyte glycoprotein antibody‐associated disease; MRI, magnetic resonance imaging.

During follow‐up, only four out of the 42 (9.5%) seropositive patients had additional ON attacks, and only one of them showed a clinical supporting feature (disc edema). Four (23.5%) CP and two (8.0%) LP patients had any supporting MRI features at any attack (p = 0.202). Overall, 12 (70.6%) CP and four (16.0%) LP cases met any supporting clinical or MRI feature at any time during the disease course (p = 0.001) (Table 3).

### 3.3 Correlation Between Qualitative (MOG‐IgG Titres) and Quantitative Measures

**FIGURE 4:**
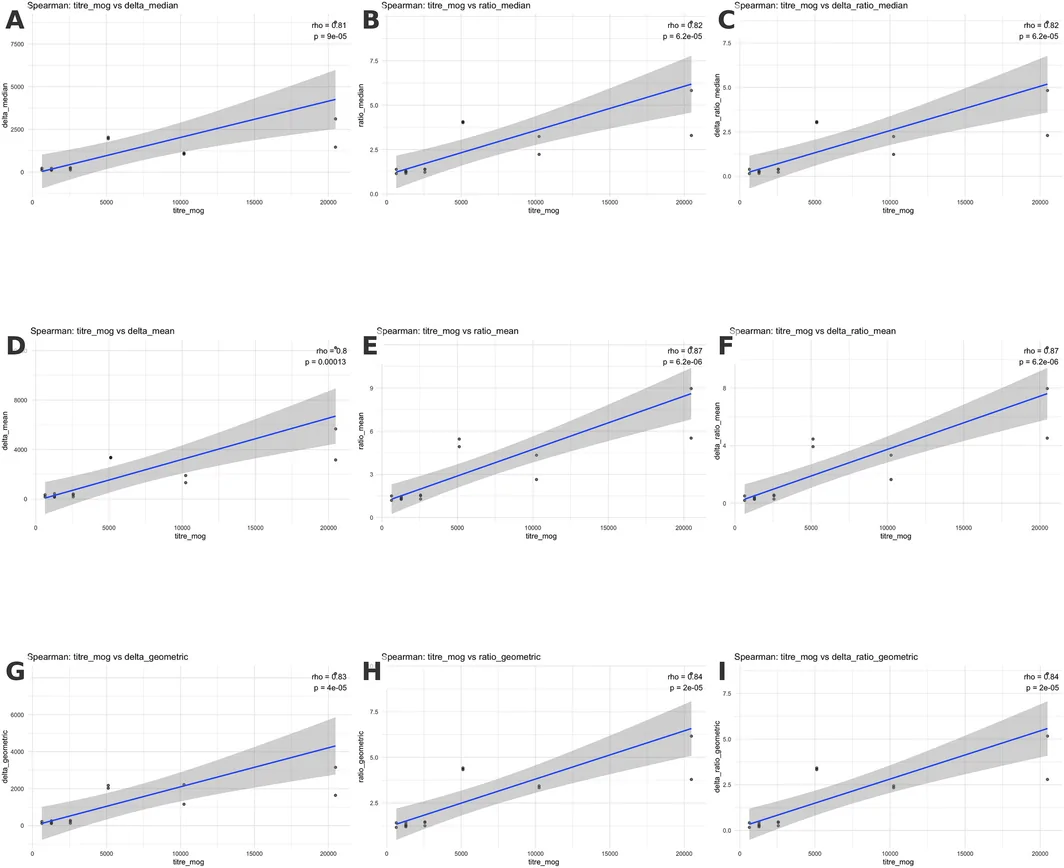
Correlation between the different quantitative measures and MOG‐IgG titres Black dots represent each CP patient included in the correlation analyses.

All the quantitative FI formulas showed strong correlation with MOG‐IgG titres. Among them, the mean delta‐ratio demonstrated the highest degree of correlation (Rho 0.87, *p* < 0.001). (Figure 4).

When assessing the positivity thresholds derived from the control cohort, the mean + 4SD mean delta‐ratio (0.17) showed the best performance for predicting MOG‐IgG positivity ≥ 1:640, yielding an area under the curve (AUC) of 0.90 [95% CI 0.82–0.99], with 81% sensitivity [58.1%–94.6%] and 100% specificity [99.4%–100%].

In addition, the Youden index was set at 0.17, thus resulting in similar performance for MOG‐IgG positivity ≥ 1:640 prediction. Based on this cutoff value, 21 patients had a quantitative MOG‐IgG positive result, 17 of whom were also positive at ≥ 1:640 by qualitative assessment. Among the four additional patients identified, one was MOG‐IgG positive at 1:160, who had a diagnosis of MS. The other three patients were negative for MOG‐IgG at low titres. These included two patients presenting with monophasic unilateral optic neuritis (with five and seven years of follow‐up, respectively) with disc edema, normal brain MRI and positive CSF‐OBs; and one patient with a monophasic brainstem syndrome (with nine years of follow‐up) with T2‐lesions not fulfilling the McDonald criteria and negative CSF‐OBs, but without supporting features of MOGAD. Clinical information for these three cases is detailed in the Supporting Information.

## 4 Discussion

In this prospective cohort of 613 adults with a first demyelinating event suggestive of MS, we identified 25 (4.08%) patients with LP MOG‐IgG. These patients were clinically indistinguishable from seronegative cases and showed similar rates of MS diagnosis during follow‐up. In contrast, CP MOG‐IgG patients differed markedly, with a higher proportion of ON, a lower proportion of CSF‐OBs, and fewer MS diagnoses. Importantly, this study reflects a real‐world MS‐predominant clinical setting, in which MOG‐IgG testing is increasingly performed despite a relatively low pretest probability of MOGAD.

In a recent study, we identified 2.7% of MOG‐IgG CP positivity (cut off ≥ 1:640) among adult patients with a first demyelinating event suggestive of MS [3]. However, it remains unclear whether “true” MOGAD cases may be missed at lower titres. Based on our live CBA‐FACS assay and following the recommendations of the MOGAD criteria [1], results are dichotomized as either CP or negative, precluding the use of supporting features in cases with low titres. In the current study, we addressed this gap by assessing MOG‐IgG at a lower dilution (1:160) in order to define an LP subgroup (MOG‐IgG positive at 1:160–1:320). Among the 613 patients in the total cohort, we identified 25 LP cases, representing a prevalence of 4.1%. Notably, LP patients were indistinguishable from seronegative cases in terms of baseline clinical and paraclinical features while CP patients showed clear differences, with a higher frequency of ON and the absence of CSF‐OBs at presentation compared to both seronegative and LP groups. Moreover, the 2017 McDonald criteria were met by most seronegative and LP patients at onset as compared with only a small proportion of CP cases.

Although the application of the MOGAD diagnostic criteria is primarily based on cohorts enriched with patients presenting with an NMOSD‐like phenotype [9, 10, 11], our findings provide additional evidence regarding the interpretation of LP MOG‐IgG results in MS‐predominant settings, which represent a common real‐world clinical scenario in Western demyelinating disease centres. Importantly, MOG‐IgG testing should be guided by clinical suspicion and interpreted within the appropriate phenotypic context.

### 4.1 The Importance of Supporting Criteria

A higher proportion of CP (65%) than LP patients (16%) had at least one supporting feature of MOGAD at the first attack. This difference was mainly driven by clinical features (i.e., a higher frequency of disc edema in CP patients). In addition, MRI supporting features were two‐fold more frequent in the CP group compared to the LP group. Although this difference did not reach statistical significance, it appears clinically meaningful, and the limited sample size should be considered when interpreting these findings. As the MOGAD diagnostic criteria do not specify whether supporting features must be present at disease onset or can also be considered during subsequent attacks, some LP patients may have been diagnosed later in the disease course despite not meeting the criteria at first presentation. In our cohort, the proportion of patients with supporting features at any point during the follow‐up was very similar compared to the first attack in both CP and LP groups, suggesting a limited impact of this uncertainty on clinical diagnosis, as suggested by other groups [9, 10, 11].

### 4.2 Time to Sampling Matters Significantly to Diagnose MOGAD

Another relevant factor for MOGAD diagnosis is the timing of sampling, as MOG‐IgG titres tend to decline over time and may become undetectable [12, 13]. Indeed, two recent studies showed that a CP result was more likely when testing was performed closer to the clinical attack [9, 13]. In our cohort, all samples were collected within 6 months of the first attack, and no significant differences in the median time from attack to sampling were observed between groups, which emphasizes the low probability of false‐negative or LP results produced by MOG‐IgG titres by a decline in MOG‐IgG titres in our cohort. Further studies with longitudinal serum assessment are needed to confirm the MOG‐IgG dynamics and their relevance to MOGAD diagnosis.

### 4.3 MS And MOGAD Overlap Cases

Two‐thirds of both seronegative and LP patients met the 2017 McDonald criteria at the last follow‐up, in contrast to 18% of CP cases. Among the 16 LP patients fulfilling the McDonald criteria, three (19%) had at least one MOGAD supporting feature, thus meeting both MS and MOGAD criteria. The potential coexistence of MOGAD and MS remains an area of ongoing debate. Although current diagnostic criteria consider both entities to be mutually exclusive, increasing cases with overlapping features have been reported, raising the possibility of dual pathology or even a spectrum between both conditions [1, 3, 14, 15].

In our cohort, patients fulfilling features suggestive of both conditions were ultimately classified as having MS based on well‐established markers, including CSF‐OBs, typical MRI lesion distribution, accrual of silent lesions, and treatment response. While we cannot completely exclude coexistence in rare cases, our findings suggest that in MS‐predominant settings, LP MOG‐IgG results are more likely to reflect false‐positive findings or epiphenomena rather than true dual pathology. Further studies are needed to better define this potential overlap and to explore whether these conditions may represent a spectrum rather than entirely distinct entities.

Overall, decreasing the positivity threshold from 1:640 (CP) to 1:160 (LP) diagnosed an additional true MOGAD case out of the 25 patients. Among the remainder, 16 had a diagnosis of MS, and eight did not fulfill a definite diagnosis. Whether these eight cases represent false‐positive results or true MOGAD cases remains uncertain. It is possible that technical limitations—such as the lack of dedicated orbital MRI or fundoscopy, or the well‐recognized radiological lag in MOGAD—may have contributed to the absence of diagnostic criteria in these patients [16]. Moreover, the recent identification of new MOG‐IgG–associated clinical entities, such as aseptic meningitis—which may present with normal MRI findings—further broadens the phenotypic spectrum of MOGAD and poses a challenge to the current diagnostic criteria [17].

### 4.4 Different Assays

Besides these clinic‐radiological barriers, the lack of standardized assays complicates the interpretation of MOG‐IgG results. Importantly, definitions of LP and CP MOG‐IgG results are not standardized and vary across laboratories depending on assay methodology, screening dilution, and interpretation criteria, which limits direct comparison of titres between studies [1]. In particular, among centers using CBA‐FC, there is marked heterogeneity in how results are evaluated. Some laboratories rely on qualitative assessment of binding [2, 3], whereas others apply quantitative approaches, such as calculating the delta (Δ) or the ratio of FI between transfected and non‐transfected cells [4, 18, 19]. However, these quantitative thresholds are often arbitrarily defined or derived from control populations [1, 4, 5]. We found a high correlation between the qualitative assessment (MOG‐IgG titres) and the quantitative formulas, especially the mean Δ‐ratio FI.

When using the best quantitative cutoff of 0.17 for predicting MOG‐IgG positivity (≥ 1:640), 21 MOG‐IgG positive cases were identified, including the 17 positive cases with the qualitative test (MOG‐IgG ≥ 1:640). Among the four additional seropositive cases, two met the MOGAD criteria (monophasic ON with disc edema) but were negative by the qualitative assessment, one had MS and was MOG‐IgG positive at 1:160, and the other had a monophasic brainstem syndrome with a negative qualitative result, fulfilling neither MOGAD nor the MS criteria. Larger studies are needed to determine whether quantitative approaches provide incremental value over qualitative titer interpretation.

### 4.5 Limitations

This study has several limitations. First, a low proportion of both LP and CP groups had supporting MRI features of MOGAD at the first attack and during follow‐up. Although initially unexpected, this likely reflects the MS‐predominant design of our cohort, in which alternative diagnoses other than MS were excluded at inclusion and reassessed during follow‐up [20]. Indeed, the aim of this study was to evaluate MOG‐IgG interpretation in real‐world clinical settings. In addition, the high proportion of ON, especially in the CP group, and the unavailability of dedicated orbital MRI sequences may explain these findings. This limitation is common across historical cohorts, as optic nerve sequences have not been systematically performed until recent years [10, 21]. Furthermore, the relatively low proportion of relapsing MOGAD cases may also reflect the specific characteristics of this MS‐predominant cohort, which may differ from previously reported MOGAD‐enriched populations, as well as the variable and, in some cases, limited duration of follow‐up.

Second, although the time from clinical onset to serum sampling was within 6 months in all cases, longitudinal data on antibody persistence and fluctuations were not available, limiting the assessment of MOG‐IgG dynamics over time. Third, while our findings suggest the utility of a quantitative fluorescence‐based threshold, the 4 SD delta‐ratio cutoff was derived from a relatively small control group, and external validation in larger independent cohorts is warranted. Finally, the study was conducted at a single center using a specific in‐house live CBA‐FC protocol, which may limit generalizability to other laboratories using different assay platforms or methodologies.

## 5 Conclusion

Overall, lowering the CBA‐FC positivity threshold to ≥ 1:160 identified only one additional true MOGAD case in this cohort of first demyelinating events suggestive of MS, while most low‐positive cases were ultimately diagnosed with MS. These findings highlight the importance of careful interpretation of low‐positive MOG‐IgG results in MS‐predominant clinical settings.

## Author Contributions

Javier Villacieros‐Álvarez, Carmen Espejo, and Álvaro Cobo‐Calvo contributed to the conception and design of the study. All authors contributed to the acquisition and analysis of data. Javier Villacieros‐Álvarez and Álvaro Cobo‐Calvo contributed to drafting the manuscript and preparing the figures.

## Funding

This study was funded by Instituto de Salud Carlos III through project PI24/00859, awarded to A.C.‐C. and co‐funded by the European Union. Additional funding was provided by The Sumaira Foundation through the SPARK Grant (TSF_SPARK_2023_03, 2023).

## Conflicts of Interest

The authors declare no conflicts of interest.

## Supporting information


**Table S1:** Chronic treatment in LP and CP patients.

**Data S1:** Clinical description of “low‐positive” cases with overlapping MS‐MOGAD features and final diganosis of MS.
Clinical description of additional MOG‐IgG positive cases by quantitative analysis.

## Data Availability

The data that support the findings of this study are available from the corresponding author upon reasonable request.

## References

[1] B. Banwell , J. L. Bennett , R. Marignier , et al., “Diagnosis of Myelin Oligodendrocyte Glycoprotein Antibody‐Associated Disease: International MOGAD Panel Proposed Criteria,” Lancet Neurology 22, no. 3 (2023): 268–282, 10.1016/S1474-4422(22)00431-8. 36706773

[2] E. Sechi , M. Buciuc , S. J. Pittock , et al., “Positive Predictive Value of Myelin Oligodendrocyte Glycoprotein Autoantibody Testing,” JAMA Neurology 78, no. 6 (2021): 741–746, 10.1001/jamaneurol.2021.0912. 33900394 PMC8077043

[3] J. Villacieros‐Álvarez , C. Espejo , G. Arrambide , et al., “Myelin Oligodendrocyte Glycoprotein Antibodies in Adults With a First Demyelinating Event Suggestive of Multiple Sclerosis,” Annals of Neurology 95 (2024): 116–128, 10.1002/ana.26793. 37705507

[4] F. Tea , D. Pilli , S. Ramanathan , et al., “Effects of the Positive Threshold and Data Analysis on Human MOG Antibody Detection by Live Flow Cytometry,” Frontiers in Immunology 11 (2020): 119, 10.3389/fimmu.2020.00119. 32117270 PMC7016080

[5] M. Reindl , K. Schanda , M. Woodhall , et al., “International Multicenter Examination of MOG Antibody Assays,” Neurol Neuroimmunol Neuroinflamm 7, no. 2 (2020): e674, 10.1212/NXI.0000000000000674. 32024795 PMC7051197

[6] A. Cobo‐Calvo , P. Carbonell‐Mirabent , C. Tur , et al., “Age‐Related Disability Outcomes After a First Demyelinating Event,” Neurology 104, no. 4 (2025): e210305, 10.1212/WNL.0000000000210305. 39903901

[7] M. P. Wattjes , O. Ciccarelli , D. S. Reich , et al., “2021 MAGNIMS‐CMSC‐NAIMS Consensus Recommendations on the Use of MRI in Patients With Multiple Sclerosis,” Lancet Neurology 20, no. 8 (2021): 653–670, 10.1016/S1474-4422(21)00095-8. 34139157

[8] A. J. Thompson , B. L. Banwell , F. Barkhof , et al., “Diagnosis of Multiple Sclerosis: 2017 Revisions of the McDonald Criteria,” Lancet Neurology 17, no. 2 (2018): 162–173, 10.1016/S1474-4422(17)30470-2. 29275977

[9] E. Fonseca , G. Olivé‐Cirera , E. Martinez‐Hernandez , et al., “Investigating the 2023 MOGAD Criteria in Children and Adults With MOG‐Antibody Positivity Within and Outside Attacks,” Neurology 103, no. 6 (2024): e209682, 10.1212/WNL.0000000000209682. 39190859

[10] S. Carta , E. Sechi , A. Dinoto , et al., “Real‐Life Evaluation of the MOGAD Diagnostic Criteria: Application Challenges and Discrepancies,” Neurol Neuroimmunol Neuroinflamm 12, no. 5 (2025): e200456, 10.1212/NXI.0000000000200456. 40829090 PMC12366032

[11] A. G. Filippatou , Y. Said , H. Chen , E. S. Vasileiou , G. Ahmadi , and E. S. Sotirchos , “Validation of the International MOGAD Panel Proposed Criteria: A Single‐Centre US Study,” Journal of Neurology, Neurosurgery, and Psychiatry 95, no. 9 (2024): 870–873, 10.1136/jnnp-2023-333227. 38569875 PMC11330367

[12] P. Waters , G. Fadda , M. Woodhall , et al., “Serial Anti‐Myelin Oligodendrocyte Glycoprotein Antibody Analyses and Outcomes in Children With Demyelinating Syndromes,” JAMA Neurology 77, no. 1 (2020): 82–93, 10.1001/jamaneurol.2019.2940. 31545352 PMC6763982

[13] M. Forcadela , C. Rocchi , D. San Martin , et al., “Timing of MOG‐IgG Testing Is Key to 2023 MOGAD Diagnostic Criteria,” Neurol Neuroimmunol Neuroinflamm 11, no. 1 (2024): e200183, 10.1212/NXI.0000000000200183. 37977848 PMC10758949

[14] H. Y. Seok , “MOG Antibody‐Positive Patients Meeting Diagnostic Criteria for MS: Is It MOGAD With an MS‐Like Phenotype or True MS?,” Neurological Sciences 46, no. 8 (2025): 3491–3494, 10.1007/s10072-025-08194-8. 40266423

[15] M. Levy , E. A. Yeh , C. H. Hawkes , J. Lechner‐Scott , and G. Giovannoni , “Implications of Low‐Titer MOG Antibodies,” Multiple Sclerosis and Related Disorders 59 (2022): 103746, 10.1016/j.msard.2022.103746. 35320765

[16] L. Cacciaguerra , O. Abdel‐Mannan , D. Champsas , et al., “Radiologic Lag and Brain MRI Lesion Dynamics During Attacks in MOG Antibody‐Associated Disease,” Neurology 102, no. 10 (2024): e209303, 10.1212/WNL.0000000000209303. 38710000 PMC11177594

[17] A. Aboseif , N. N. Kim , G. Bou , et al., “Meningitis as an Attack Phenotype of Myelin Oligodendrocyte Glycoprotein Antibody‐Associated Disease,” JAMA Neurology 82, no. 8 (2025): 871–873, 10.1001/jamaneurol.2025.1774. 40522676 PMC12340653

[18] A. Cobo‐Calvo , M. Sepúlveda , H. d'Indy , et al., “Usefulness of MOG‐Antibody Titres at First Episode to Predict the Future Clinical Course in Adults,” Journal of Neurology 266, no. 4 (2019): 806–815, 10.1007/s00415-018-9160-9. 30607536

[19] J. A. Lopez , S. D. Houston , F. Tea , et al., “Validation of a Flow Cytometry Live Cell‐Based Assay to Detect Myelin Oligodendrocyte Glycoprotein Antibodies for Clinical Diagnostics,” Journal of Applied Laboratory Medicine 7, no. 1 (2022): 12–25, 10.1093/jalm/jfab101. 34718586

[20] A. Vilaseca , M. Tintoré , P. Carbonell‐Mirabent , et al., “Uncovering Alternative Diagnoses in Patients With Clinical Syndromes Suggestive of Multiple Sclerosis: A Transversal Study From the Prospective Barcelona CIS Cohort,” Multiple Sclerosis 31, no. 4 (2025): 408–417, 10.1177/13524585251314749. 39907218

[21] J. Sastre‐Garriga , A. Vidal‐Jordana , A. T. Toosy , et al., “Value of Optic Nerve MRI in Multiple Sclerosis Clinical Management: A MAGNIMS Position Paper and Future Perspectives,” Neurology 103, no. 3 (2024): e209677, 10.1212/WNL.0000000000209677. 39018513 PMC11271394

