# Supplementary material for: “Low‐Positive” MOG‐IgG Cases Among Adults With a First Event Suggestive of Multiple Sclerosis
Source: Ann Clin Transl Neurol. 2026 Aug 21:10.1002/acn3.70496. Online ahead of print. doi: 10.1002/acn3.70496 (PMC13498885; doi:10.1002/acn3.70496)
Supplement: Supplementary file 1 [file ACN3-9999-0-s001.docx]

**Table S1. Chronic treatment in LP and CP patients**

|  | **LP patients**  **(n=25)** | **CP patients (n=17)** | **P-value** |
| --- | --- | --- | --- |
| **Maintenance treatment; n (%)** | 14 (56.0) | 4 (8.3) | **<0.001** |
| **First-line treatment** | Interferon beta (N=7), glatiramer acetate (N=2), dimethyl fumarate (N=2), clinical trial (N=3) | Interferon beta (N=2), glatiramer acetate (N=1), azathioprine (N=1) |  |
| **Second-line treatment** | Glatiramer acetate (N=2), dimethyl fumarate (N=2), fingolimod (N=1), ofatumumab (N=1) | NA |  |
| **Third-line treatment** | Dimethyl fumarate (N=1) | NA |  |
