# Supplementary material for: “Low‐Positive” MOG‐IgG Cases Among Adults With a First Event Suggestive of Multiple Sclerosis
Source: Ann Clin Transl Neurol. 2026 Aug 21:10.1002/acn3.70496. Online ahead of print. doi: 10.1002/acn3.70496 (PMC13498885; doi:10.1002/acn3.70496)
Supplement: Supplementary file 2 [file ACN3-9999-0-s002.docx]

**Clinical description of “low-positive” cases with overlapping MS-MOGAD features and final diganosis of MS**

**# Patient 1**

A 31-year-old female with a history of bilateral uveitis (pars planitis, 2007) presented in 2012 with a left optic neuritis. Ophthalmological examination showed a visual acuity of 0.4 and optic disc edema in the left eye (**supporting clinical feature**). Baseline MRI revealed T2 lesions in subcortical, periventricular, corpus callosum, brainstem, and cervical spinal cord locations, fulfilling DIS criteria, with no gadolinium-enhancing lesions. CSF analysis demonstrated CSF-restricted oligoclonal bands and an elevated IgG index (1.09). Alternative etiologies were excluded, thus meeting McDonald criteria for MS.

Glatiramer acetate was initiated 4 months after the first attack. A follow-up MRI 8 months later showed three new T2 lesions—two of them contrast-enhancing. Another MRI 4 months afterward demonstrated one new gadolinium-enhancing T2 lesion.

In 2015, glatiramer acetate was switched to dimethyl fumarate due to intolerance and radiological activity. Since then, the patient has remained free of relapses and MRI activity. At the last follow-up in 2024, EDSS was 1.0.

**# Patient 2**

A 26-year-old female with no significant past medical history presented in 2020 with a left optic neuritis. Ophthalmological examination showed a visual acuity of counting fingers at 40 cm, without disc edema. Baseline brain MRI revealed a long T2 lesion involving the posterior segment of the left optic nerve with intense contrast enhancement (**supporting MRI feature**), as well as subcortical, corpus callosum, brainstem, and middle cerebellar peduncle lesions. Spinal cord MRI was normal.

CSF analysis showed absence of oligoclonal bands and a normal IgG index (0.5), and blood tests were unremarkable. Visual acuity improved dramatically after intravenous methylprednisolone, reaching visual acuity 1.0.

A follow-up MRI one year after the attack showed no new lesions. However, a subsequent MRI in 2022 revealed two new silent periventricular T2 lesions, meeting both DIS and DIT criteria for MS. Dimethyl fumarate was initiated in 2023.

At last follow-up in 2024, the patient remained free of relapses and radiological activity, with an EDSS of 1.5.

**# Patient 3**

A 23-year-old female with no relevant medical history presented in 2003 with a partial spinal cord syndrome with a cervical sensory level (EDSS 2.5). She experienced partial recovery after intravenous methylprednisolone. Baseline MRI revealed T2 lesions in the spinal cord at C1–C2, and supratentorial juxtacortical and periventricular lesions, including one gadolinium-enhancing lesion, fulfilling both DIS and DIT criteria. CSF analysis was not performed.

Interferon beta-1a was initiated 5 months after the attack. Six months after treatment initiation, she experienced a relapse (myelitis) but treatment was maintained. In 2013, follow-up MRI showed 14 new silent T2 lesions; however, due to the absence of clinical relapses or disability progression, therapy was not escalated.

In 2023, she experienced a new relapse with spinal cord involvement. Spinal MRI confirmed a T2 lesion in the conus medullaris (**supporting MRI feature**) with gadolinium enhancement, while brain MRI showed no new silent lesions. Ofatumumab was subsequently started.

At last follow-up in 2024, EDSS was 3.5.

**Clinical description of additional MOG-IgG positive cases by quantitative analysis**

**# Patient 1**

A 34-year-old male with medical history of rosacea presented in 2013 with a left optic neuritis. Opthalmological examination showed a visual acuity of 0.2, and optic disc edema in the left eye (**supporting clinical feature**). Visual acuity improved from 0.2 to 0.8 after intravenous methylprednisolone.

Baseline brain MRI showed a unique T2-lesion located in corpus callosum, thus not meeting DIS criteria. CSF analysis revealed the presence of CSF-restricted oligoclonal bands, and blood tests were normal.

At last follow-up in 2020, the patient remained free of relapses and radiological activity (last MRI in 2018 without changes), and EDSS was 1.0.

**# Patient 2**

A 32-year-old male with no significant past medical history presented in 2007 with left optic neuritis. Ophthalmological examination revealed a visual acuity of hand movement, and optic disc edema (**supporting clinical feature**). Visual acuity recovered to 1.0 after intravenous methylprednisolone.

Baseline brain MRI was normal. CSF analysis demonstrated the presence of CSF-restricted oligoclonal bands. Blood tests did not show relevant findings.

At last follow-up in 2012 the patient was free of relapses and radiological activity (last brain MRI in 2012 was normal), and EDSS was 0.0.

**# Patient 3**

A 44-year-old female with no significant past medical history presented in 2006 with a partial myelitis. Baseline brain and spinal cord showed typical MS T2-lesions meeting DIS, and CSF analysis revealed the presence of CSF-restricted oligoclonal bands. In 2009 she presented with a new partial myelitis, and follow-up MRI demonstrated 5 new T2-lesions. Interferon beta-1a was initiated in 2009. In 2012 she was diagnosed with anterior uveitis in right eye requiring topic corticosteroids.

At last follow-up in 2025 she has remained free of relapses and with only 3 new T2-lesions accumulated on serial brain MRIs (last MRI in 2024). EDSS was 1.5.

**# Patient 4**

A 38-year-old female with no significant past medical history presented in 2010 with right facial numbness, with no other neurological symptoms or signs on examination. Baseline brain MRI showed subcortical and periventricular T2-lesions not fulfilling DIS, and spinal cord MRI was normal. CSF analysis showed absence of CSF-restricted oligoclonal bands, and blood tests were unremarkable.

At last follow-up in 2023 the patient was free of relapses and radiological activity (last brain MRI in 2020 was normal), and EDSS was 0.0.
